# Lack of association between proton pump inhibitor use and brain aging: a cross-sectional study

**DOI:** 10.1007/s00228-020-03068-8

**Published:** 2021-01-13

**Authors:** Nayeon Ahn, Stefan Frenzel, Katharina Wittfeld, Robin Bülow, Henry Völzke, Markus M. Lerch, Jean-Francois Chenot, Ulf Schminke, Michael Nolde, Ute Amann, Christa Meisinger, Jakob Linseisen, Sebastian E. Baumeister, Hans Jörgen Grabe, Ina-Maria Rückert-Eheberg

**Affiliations:** 1 Chair of Epidemiology, Ludwig-Maximilians-Universität München, UNIKA-T Augsburg, Neusässer Str. 47, 86156 Augsburg, Germany; 2 Independent Research Group Clinical Epidemiology, Helmholtz Zentrum München, German Research Center for Environmental Health (GmbH), Neuherberg, Germany; 3 Department of Psychiatry and Psychotherapy, University Medicine Greifswald, Greifswald, Germany; 4 German Center for Neurodegenerative Diseases (DZNE), Greifswald/Rostock, Site Greifswald, Greifswald, Germany; 5 Institute for Diagnostic Radiology and Neuroradiology, University Medicine Greifswald, Greifswald, Germany; 6 Institute for Community Medicine, University Medicine Greifswald, Greifswald, Germany; 7 Department of Medicine A, University Medicine Greifswald, Greifswald, Germany; 8 Department of Neurology, University Medicine Greifswald, Greifswald, Germany; 9 Institute of Epidemiology, Helmholtz Zentrum München, German Research Center for Environmental Health (GmbH), Neuherberg, Germany

**Keywords:** Dementia, Cognitive impairment, Proton pump inhibitors, Brain volume, Magnetic resonance imaging

## Abstract

**Purpose:**

Due to conflicting scientific evidence for an increased risk of dementia by intake of proton pump inhibitors (PPIs), this study investigates associations between PPI use and brain volumes, estimated brain age, and cognitive function in the general population.

**Methods:**

Two surveys of the population-based Study of Health in Pomerania (SHIP) conducted in Northeast Germany were used. In total, 2653 participants underwent brain magnetic resonance imaging (MRI) and were included in the primary analysis. They were divided into two groups according to their PPI intake and compared with regard to their brain volumes (gray matter, white matter, total brain, and hippocampus) and estimated brain age. Multiple regression was used to adjust for confounding factors. Cognitive function was evaluated by the Verbal Learning and Memory Test (VLMT) and the Nuremberg Age Inventory (NAI) and put in relation to PPI use.

**Results:**

No association was found between PPI use and brain volumes or the estimated brain age. The VLMT score was 1.11 lower (95% confidence interval: − 2.06 to − 0.16) in immediate recall, and 0.72 lower (95% CI: − 1.22 to − 0.22) in delayed recall in PPI users than in non-users. PPI use was unrelated to the NAI score.

**Conclusions:**

The present study does not support a relationship between PPI use and brain aging.

**Supplementary Information:**

The online version contains supplementary material available at 10.1007/s00228-020-03068-8.

## Introduction

Much attention in the medical and scientific communities has been paid to suspected associations of proton pump inhibitors (PPIs) with adverse effects, since PPIs are widely used for gastric acid–related disorders, often over-prescribed and sold over the counter [1, 2]. In view of the fact that dementia is a common and burdensome disease in aging societies, it is crucial to identify avoidable risk factors such as specific pharmaceutical agents [3].

Although plausible pathophysiological pathways of brain deterioration that PPIs might be involved in have been described [4], previous researches have revealed conflicting evidence for a link between PPI use and the risk of dementia and cognitive decline [5–7].

The studies to date have mostly relied on clinical diagnoses [8] or neuropsychological tests [9] to define dementia or cognitive impairment, which are prone to misclassification errors [10]. In the present study, we conducted an analysis of PPI use in relation to brain volumes and estimated brain age derived from magnetic resonance imaging (MRI) [11–13]. We also evaluated the association between PPI use and cognitive function.

## Methods

### Study population

Data were drawn from the Study of Health in Pomerania (SHIP), that consists of two independent samples of adults from a northeastern German region. Among the original sample of 7008 individuals (SHIP-0), 2333 individuals remained at the third examination cycle (SHIP-2), and the follow-up examination was conducted between 2008 and 2012. Concurrent with SHIP-2, a new age- and sex-stratified random sample, SHIP-Trend, of 8826 individuals was drawn and 4420 (2275 women) participated. Examinations for SHIP-Trend were conducted from 2008 to 2012. More details about the study designs, recruitment, and procedures have been published elsewhere [14].

**Fig. 1:**
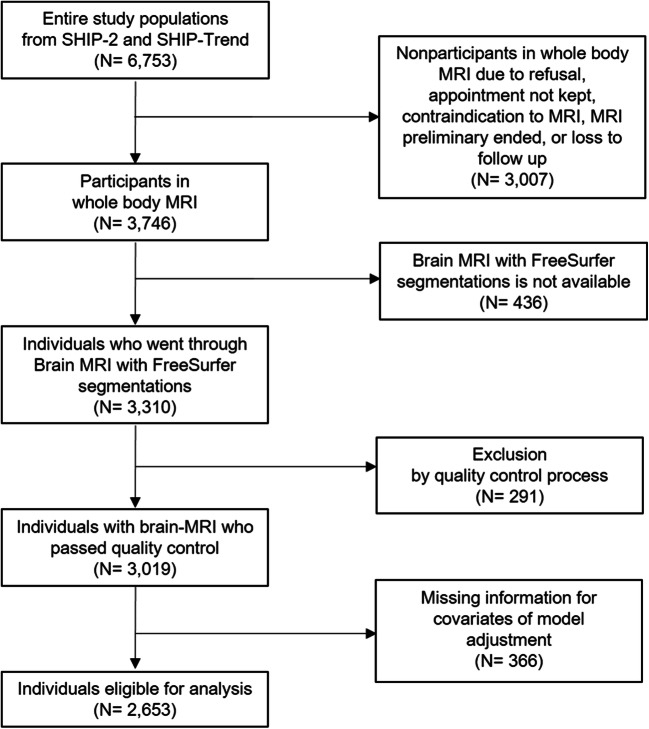
Flow chart of the MRI study population selection. MRI, magnetic resonance imaging

Individuals from SHIP-2 and SHIP-Trend were invited to participate in whole-body MRI; 3746 individuals participated in whole-body MRI [15]; 3310 participants aged 21–89 years were examined for brain MRI with FreeSurfer segmentations. Among them, individuals with MRI scans that did not pass quality control (e.g., inhomogeneity check of the magnetic field or severe movement artifacts) (*n* = 291) or with missing information (*n* = 366) were excluded. As a result, the analytic cohort for the analysis of PPI intake and MRI-derived outcome variables comprised 2653 participants (SHIP-2 = 788, SHIP-Trend = 1865) (Fig. 1). For the analysis on verbal memory assessments, data from 5711 study participants (SHIP-2: 1569, SHIP-Trend: 4142) were included.

The Ethics Committee of the University Greifswald approved the study protocols of SHIP and SHIP-Trend. All participants provided their written informed consent.

### Assessment of PPI use

Medications taken during the last 7 days were assessed within an interview using the name of the drug product or the unequivocal drug package code. This information was then used to identify the active substances and translate this into the Anatomical Therapeutic Chemical (ATC) code for further investigation. Additional questions focused on the drug use pattern by discriminating between “regular use” and “use on demand.” PPI use was defined as “regular use” (yes/no) including omeprazole, pantoprazole, lansoprazole, rabeprazole, and esomeprazole (ATC codes A02BC01-05).

### Measurement of brain volumes

The neurocranium unit of the SHIP-MRI included a T1-weighted and fluid-attenuated inversion recovery (FLAIR) sequence. MRI scans were obtained using a 1.5 Tesla MRI machine (Magnetom Avanto, Siemens Medical Systems, Erlangen, Germany). The T1-weighted images were acquired with the following parameters: slice thickness = 1.0 mm (flip angle 15 °), 3.4 ms echo-time, 1900 ms repetition time, and a voxel size of 1.0 × 1.0 × 1.0 mm^3^ [16]. Images were analyzed by the fully automated and validated segmentation software FreeSurfer version 5.3 [17]. In this study, we examined the volumes of the hippocampus (left, right, and sum of both sides, respectively), gray matter, white matter, and the total brain. The total brain volume was calculated as the sum of gray matter volume and white matter volume.

### Assessment of the estimated brain age

Cortical reconstruction and volumetric segmentation were performed with the FreeSurfer image analysis suite version 5.3. In total, 169 brain regions of gray matter, white matter, and the ventricular system were considered for the estimation of the brain age. Brain ages were calculated using sex-stratified ridge regression models of chronological age on the volumes of all 169 brain regions. More specifically, the brain age of an individual was defined as his predicted age using a model based on all 169 regional brain volumes from the remaining individuals of the same sex. A similar approach has recently been used successfully to predict the presence of Alzheimer’s disease based on MRI images [16]. The complete list of brain regions used for estimating brain age can be found in the supplementary information of the previous study [16]. The corresponding sex-specific coefficients of our brain age model can be provided by the corresponding author upon request.

### Verbal memory tests

To assess the verbal memory of the study participants, a slightly abridged version of the Verbal Learning and Memory Test (VLMT), the German version of the Rey auditory-verbal learning test [18], was conducted in SHIP-2. It consisted of consecutive learning of a list of 15 words over three trials with immediate recall after each trial. After the three trials were finished, a second word list was given to the participants without previous notice to include the effects of interference. After 20 min, the participants were asked to recall the first word list. The sum of correctly recalled words from the three immediate recall trials reflects short-term and working memory (max. 45 points). The sum of correctly recalled words after 20 min was used as a measure of delayed recall (max. 15 points) [19].

The Nuremberg Age Inventory (NAI) was carried out in SHIP-Trend. The NAI is a German collection of tests and questionnaires devised to assess the cognitive abilities during brain aging [20]. It includes subsets of verbal learning and memory and consists of eight words. The participants were asked to recall as many words as possible immediately after hearing the eight words. After 20 min, the participants were asked to retrieve them, mixed with eight additional distractor words. The sum score is defined as a sum of the number of correctly identified words minus the number of falsely chosen distractor words (max. 8 points).

### Confounders

We controlled for several confounders, assuming that direct causes of the exposure or outcome, excluding possible instrumental variables, would identify a sufficient set of confounding variables [21]. Because of the multi-origins of the different types of dementia (e.g., dementia with Alzheimer’s diseases and vascular dementia), it is complicated to consider all socio-demographic and clinical characteristics, including genetic factors, that could increase the risk of dementia in PPI users. The associations of brain volumes with socio-demographic factors, e.g., education level [22] and income [23] or behavioral factors such as smoking [24] and alcohol consumption [25] have been well-described in previous studies. Since obesity plays a critical role as a confounder [26, 27], we considered the body mass index (BMI) for model adjustment. In addition, we explored several drug classes known as cognitive function–altering medications [28, 29]. Also, we investigated the medicines that are frequently taken together with PPIs [30, 31].

Socio-demographic variables, medical history, and clinical data were collected through a standardized computer-assisted face-to-face interview [14]. At baseline, income was adjusted by dividing the household income by the square root of the number of household members. The clinical data used in the current study have been described in more detail elsewhere [12]. Specifically, we included the following covariables for adjustment: age; sex; intracranial volume (assessed by FreeSurfer 5.3); education level (< 10, = 10, > 10 years in school); smoking experience (never, former, or current smoker); income (Euros); alcohol consumption (g/day, derived from beverage-specific quantity-frequency indices); BMI (kg/m^2^); total cholesterol/high-density lipoprotein cholesterol ratio (TC/HDL-C); glycated hemoglobin (HbA1c); use of antidepressants (ATC codes: N06A*), antidiabetics (A10*), antihypertensives (C02*, C03*, C07*, C08*, C09*), anti-inflammatory medication including non-steroidal anti-inflammatory drugs (NSAIDs) (B01AC06, B01AC08, B01AC15, B01AC34, B01AC36, B01AC56, C01EB03, C01EB16, C10BX01, C10BX02, C10BX04, C10BX05, M01*, N02BA*, N02BB*, N02BG*), statins (C10A*), and anticholinergics (ATC codes based on the active substances by Gray SL et al. [28]); study (SHIP-2, SHIP-Trend); and the existence of cerebrovascular pathologies or lesions in the brain that might affect brain volumes found by brain MRI scanning during this study (yes/no, for details see Supplementary Table 1S).

### Statistical analyses

Baseline characteristics were compared between PPI users and PPI non-users by computation of medians (25th, 75th percentile) for continuous variables and percentages for categorical variables. For the primary analysis, linear regression models were used to assess the associations of PPI intake with the global volume measures of the hippocampus, gray matter, white matter, and the total brain, and the estimated brain age. In secondary analyses, we used linear regression to assess the association between PPI use and VMLT and NAI scores, respectively. Models were adjusted for the confounders described in the methods section and the interaction between age and sex. Age was included in the analysis using restricted cubic splines. The primary analysis was also adjusted for a covariable indicating the presence of a cerebrovascular pathology or a lesion in the brain. We further evaluated the modifying effects of age on PPI use for brain volumes, estimated brain age, and verbal memory tests.

Since not all SHIP participants went through the brain MRI scan, we tested the plausibility of the missing-completely-at random (MCAR) assumption underlying our primary models by fitting a multivariable logistic regression model for computing sample weights, i.e., weights for taking part in the brain MRI scan. We used inverse probability weighting (IPW) to minimize selection bias caused by non-random participation in the MRI examination [32]. IPWs were stabilized to improve precision [33]. To stabilize weights, we set the numerator of each weight equal to the marginal probability of taking part in the MRI examination.

In sensitivity analyses, we excluded study participants with the presence of cerebrovascular pathologies or lesions in the brain (*n* = 706) or did not adjust the models for the binary “brain lesions” variable. In further sensitivity analyses, we excluded participants with on-demand PPI intake (*n* = 36) from the group of the non-PPI users or excluded both, individuals with possible brain conditions and on-demand PPI users (*n* = 733).

In the secondary analysis, PPI intake was put in relation to VLMT and NAI scores using linear regression models. For model adjustment, the confounders that were used in the primary analysis were applied, except intracranial volume and the brain lesions variable. For easier comparison of the associations between PPI use and verbal memory tests, we computed standardized outcomes as well, and Cohen’s *d* was used as a measure of effect size. We additionally estimated regression models accounting for the complex sampling strategy (clustering, stratification, inverse probabilities of selection) using the R survey package. The estimates form the analyses were virtually identical (not reported). To check positivity, we used inverse probability of treatment weighting (IPTW) as a second modeling strategy, checking whether there are large weights and comparing standardized differences between PPI-exposed and unexposed individuals [34, 35]. For sensitivity analyses, we repeated the regression analyses and adjusted for all covariates but antihypertensives since a very high number of participants took antihypertensives as a comedication. The statistical software R (version 3.5.2, The R Foundation for Statistical Computing, Vienna, Austria) was used.

## Results

Of the 2653 participants in the primary analysis (21–89 years, 52.6% women), 170 (6.4%) were regular PPI users (Table 1). Compared with non-users, PPI users were older and had more cerebrovascular risk factors or brain lesions, higher BMI, and higher total cholesterol/HDL-C ratio. PPI users were more likely women.

**Table 1:** Characteristics of the MRI study population (*n* = 2653)

	PPI non-user	PPI user
	(*n* = 2483)	(*n* = 170)
SHIP-2	725	63
SHIP-Trend	1758	107
GMV (ml)	610 (568, 655)	585 (549, 618)
WMV (ml)	537 (492, 589)	522 (483, 567)
TBV (ml)	1113 (1035, 1197)	1066 (1002, 1148)
HV (ml)	7.96 (7.36, 8.54)	7.62 (7.19, 8.17)
Left HV (ml)	3.94 (3.63, 4.23)	3.77 (3.52, 4.04)
Right HV (ml)	4.03 (3.72, 4.34)	3.86 (3.61, 4.16)
ICV (ml)	1576 (1473, 1696)	1530 (1437, 1644)
Brain age (years)	52.0 (43.2, 60.0)	58.4 (50.5, 66.7)
Age (years)	51.0 (41.0, 62.0)	60.0 (50.0, 68.0)
Women (*n*,%)	1291 (52.0)	104 (61.2)
Brain lesion or vascular risk factor (n,%)	652 (26.3)	54 (31.8)
School education (*n*,%)		
< 10 years	368 (14.8)	45 (26.5)
10 years	1388 (55.9)	89 (52.4)
> 10 years	727 (29.3)	36 (21.2)
Income (€)	1255 (895, 1717)	1096 (778, 1450)
Body mass index (kg/m^2^)	27.0 (24.3, 30.2)	29.5 (26.8, 32.5)
Smoking (*n*,%)		
Never	976 (39.3)	72 (42.4)
Ex-smoker	951 (38.3)	66 (38.8)
Current	556 (22.4)	32 (18.8)
Alcohol consumption (g/day)	4.0 (1.0, 11.0)	4.0 (0.9, 9.2)
Systolic blood pressure (mmHg)	127.0 (115.0, 138.5)	130.0 (119.0, 139.0)
Diastolic blood pressure (mmHg)	77.5 (71.0, 84.0)	78.0 (73.5, 83.9)
LDL cholesterol (mmol/l)	3.3 (2.7, 4.0)	3.6 (3.0, 4.3)
HDL cholesterol (mmol/l)	1.4 (1.2, 1.7)	1.4 (1.1, 1.7)
Total cholesterol/HDL cholesterol	3.4 (2.8, 4.1)	3.6 (3.1, 4.3)
Triglycerides (mmol/l)	1.3 (0.9, 1.9)	1.7 (1.2, 2.4)
Glycated hemoglobin (%)	5.2 (4.9, 5.6)	5.4 (5.1, 5.8)
Anticholinergics (*n*,%)	46 (1.9)	13 (7.7)
Antidepressants (*n*,%)	99 (4.0)	14 (8.2)
Antidiabetic drugs (*n*,%)	94 (3.8)	13 (7.7)
Antihypertensive drugs (*n*,%)	765 (30.8)	110 (64.7)
Anti-inflammatory drugs (*n*,%)	247 (10.0)	37 (21.8)
Statins (*n*,%)	230 (9.3)	36 (21.2)

Data are medians (25th, 75th percentile) or *n* (percentages); PPI use was defined as only “regular use”

*GMV*, brain gray matter volume; *WMV*, brain white matter volume; *TBV*, total brain volume; *HV*, hippocampal volume; *ICV*, intracranial volume; *LDL*, low-density lipoprotein; *HDL*, high-density lipoprotein; *SE*, standard error; *CI*, confidence interval

PPI use was not associated with volumes of gray matter, white matter, and hippocampus (see Table 2). Similarly, PPI use was not related to brain age. The association between PPI use and right hippocampal volume was modified by age (*P* for interaction = 0.038, Supplementary Fig. 1S).

**Table 2:** Linear regression coefficients, SEs, and 95% CIs for the associations of PPI intake with brain volumes and brain age, respectively (*n* = 2653)

	Coefficient	SE	95% CI	*p*
GMV	− 1.59	2.62	(− 6.72, 3.54)	.54
WMV	2.52	3.18	(− 3.71, 8.76)	.43
TBV	0.96	3.77	(− 6.42, 8.34)	.80
HV	− 0.006	0.058	(− 0.120, 0.109)	.92
Left HV	0.009	0.030	(− 0.050, 0.069)	.76
Right HV	− 0.015	0.032	(− 0.078, 0.049)	.65
Brain age	0.67	0.60	(− 0.51, 1.85)	.26

Models are adjusted for age; sex; interaction between age and sex; intracranial volume; existence of brain lesion or vascular risk factor; education level; income; smoking; alcohol consumption; total cholesterol/HDL cholesterol ratio; glycated hemoglobin (HbA1C); systolic blood pressure; body mass index (BMI); study cohort effect; and use of anticholinergic drugs, antidepressants, antidiabetic drugs, antihypertensive drugs, anti-inflammatory drugs, and statins. Inverse probability weighting was used to correct for non-random MRI examination

*GMV*, brain gray matter volume (ml); *WMV*, brain white matter volume (ml); *TBV*, total brain volume (ml); *HV*, hippocampal volume (ml); *SE*, standard error; *CI*, confidence interval

Exclusion of the on-demand PPI users from sensitivity analysis did not change the results. Furthermore, the results were similar when study participants with the presence of cerebrovascular pathologies or lesions in the brain were excluded.

In the secondary analysis, we investigated the association of PPI intake with immediate and delayed verbal memory tests. The clinical characteristics of those participants are displayed in Table 3. We found that PPI users performed worse than non-users, with a 1.11 lower score (95% CI: − 2.06 to − 0.16) in immediate recall (score range: 0 to 45) and a 0.72 lower score (95% CI: − 1.22 to − 0.22) in delayed recall (score range: 0 to 15) assessed by VLMT. In contrast, no differences in both immediate recall (range: 0 to 8) and delayed recall (range: − 8 to 8) were observed between the two groups, when NAI was used for the cognitive assessment (see Table 4). For easier comparison of the results, standardized outcomes are also shown in the table. Furthermore, the association between PPI intake and the delayed verbal recall assessed by VLMT was modified by statin intake (*P* for interaction 0.001). Participants with combined PPI and statin intake had a 0.51 (95% CI: − 0.35 to 1.37) higher delayed verbal recall score than those who took PPIs but no statins (data not shown).

**Table 3:** Characteristics of the study population who went through verbal memory assessments (*n* = 5711)

	SHIP-2		SHIP-Trend	
	PPI non-user	PPI user	PPI non-user	PPI user
	(*n* = 1438)	(*n* = 131)	(*n* = 3855)	(*n* = 287)
VLMT				
Immediate recall (score range: 0 to 45)	26 (21, 30)	22 (20, 26)	n/a	n/a
Delayed recall (0 to 15)	8 (6, 10)	7 (5, 8)	n/a	n/a
NAI				
Immediate recall (0 to 8)	n/a	n/a	5 (4, 6)	5 (4, 6)
Delayed recall (− 8 to 8)	n/a	n/a	6 (5, 7)	6 (4, 7)
Age (years)	56.0 (45.0, 66.0)	64.0 (54.5, 71.5)	51.0 (39.0, 63.0)	63.0 (52.0, 71.5)
Women (*n*, %)	747 (51.9)	72 (55.0)	1992 (51.7)	146 (50.9)
School education (*n*, %)				
< 10 years	311 (21.6)	46 (35.1)	846 (21.9)	111 (38.7)
10 years	799 (55.6)	63 (48.1)	2021 (52.5)	123 (42.8)
> 10 years	328 (22.8)	22 (16.8)	988 (25.6)	53 (18.5)
Income (€)	1007 (701, 1356)	1086 (826, 1356)	1184 (895, 1761)	1096 (778, 1450)
Body mass index (kg/m^2^)	27.7 (24.8, 31.1)	27.8 (24.4, 31.7)	28.0 (25.0, 31.0)	28.2 (25.6, 31.6)
Smoking (*n*, %)				
Never	544 (37.8)	55 (42.0)	1391 (36.1)	107 (37.3)
Ex-smoker	609 (42.4)	59 (45.0)	1416 (36.7)	123 (42.8)
Current	285 (19.8)	17 (13.0)	1048 (27.2)	57 (19.9)
Alcohol consumption (g/day)	5.0 (2.0, 14.0)	4.1 (1.4, 13.1)	3.5 (0.7, 10..9)	2.1 (0.0, 7.5)
Systolic blood pressure (mmHg)	131.5 (119.5, 144.6)	131.0 (118.5, 142.5)	132.0 (119.0, 144.0)	134.0 (120.5, 147.5)
Diastolic blood pressure (mmHg)	80.0 (73.0, 86.5)	77.5 (71.0, 83.0)	79.0 (72.0, 86.0)	79.0 (71.5, 85.5)
LDL cholesterol (mmol/l)	3.3 (2.7, 3.9)	3.5 (2.8, 4.2)	3.3 (2.7, 4.0)	3.4 (2.7, 4.0)
HDL cholesterol (mmol/l)	1.4 (1.2, 1.7)	1.4 (1.1, 1.6)	1.4 (1.2, 1.7)	1.3 (1.1, 1.6)
Total cholesterol/HDL cholesterol	3.4 (2.8, 4.0)	3.5 (3.0, 4.3)	3.4 (2.8, 4.1)	3.5 (3.0, 4.2)
Triglycerides (mmol/l)	1.6 (1.0, 2.3)	1.8 (1.3, 2.6)	1.4 (0.9, 2.0)	1.7 (1.3, 2.6)
Glycated hemoglobin (%)	5.3 (5.0, 5.7)	5.6 (5.1, 5.9)	5.2 (4.9, 5.6)	5.5 (5.2, 6.0)
Anticholinergics (*n*, %)	36 (2.5)	14 (10.7)	81 (2.1)	13 (4.5)
Antidepressants (*n*, %)	62 (4.3)	14 (10.7)	171 (4.4)	32 (11.1)
Antidiabetic medication (*n*, %)	110 (7.7)	16 (12.2)	266 (6.9)	44 (15.3)
Anti-HTN medication (*n*, %)	600 (41.7)	89 (67.9)	1361 (35.3)	210 (73.2)
Anti-inflammatory medication (*n*, %)	213 (14.8)	46 (35.1)	472 (12.2)	101 (35.2)
Statins (*n*, %)	202 (14.0)	48 (36.6)	445 (11.5)	81 (28.2)

Data are medians (25th, 75th percentile) or *n* (percentages); PPI use was defined as only “regular use”; participants who took the verbal learning and memory assessment were included regardless of conduct of MRI examinations

*VLMT*, Verbal Learning and Memory Test; *NAI*, Nuremberg Age Inventory; *LDL*, low-density lipoprotein; *HDL*, high-density lipoprotein

**Table 4:** Linear regression coefficients, SEs, and 95% CIs for the association of PPI intake with verbal memory assessments (n = 5711)

		Unstandardized outcomes			Standardized outcomes			
		Coefficient	SE	95% CI	Coefficient	SE	95% CI	*p*
VLMT	Immediate recall	− 1.11	0.48	(− 2.06, − 0.16)	− 0.18	0.08	(− 0.34, − 0.03)	.02
	Delayed recall	− 0.72	0.26	(− 1.22, − 0.22)	− 0.24	0.08	(− 0.40, − 0.07)	.01
NAI	Immediate recall	0.01	0.07	(− 0.14, 0.15)	0.004	0.05	(− 0.10, 0.11)	.94
	Delayed recall	− 0.17	0.10	(− 0.36, 0.03)	− 0.10	0.06	(− 0.21, 0.02)	.10

Models are adjusted for age; sex; interaction between age and sex; education level; income; smoking; alcohol consumption; total cholesterol/HDL cholesterol; glycated hemoglobin (HbA1C); systolic blood pressure; body mass index (BMI); and use of anticholinergic drugs, antidepressants, antidiabetic drugs, antihypertensive drugs, anti-inflammatory drugs, and statins

*VLMT*, Verbal Learning and Memory Test (*n* = 1569); *NAI*, Nuremberg Age Inventory (*n* = 4142); *SE*, standard error; *CI*, confidence interval

In the additional analyses using IPTW, we could check that the positivity assumption is not violated from comparing standardized differences between PPI-exposed and unexposed individuals. The estimates are shown in the Supplementary Table S2. The results also suggest there is a lack of association between proton pump inhibitor use and brain aging. In the sensitivity analysis, the change in estimates of models with and without antihypertensives suggested that antihypertensive medication does not have an effect on our outcomes, although it fulfills the disjunctive cause criterion.

## Discussion

This population-based study investigated the association between PPI intake and brain aging, using brain volumes and estimated brain age as outcomes in 2653 individuals aged 21–89 years. After adjustment for multiple confounders, we did not find relations between PPI intake and brain volumes. Estimated brain age did not show a difference between PPI users and PPI non-users. Although the association between PPI use and right hippocampal volume was slightly modified by age, no significant association was found.

PPIs are valued as the most effective therapeutic agents for various conditions related to gastric acid. The prescription rates linearly increased and still ranked first among all gastrointestinal medications in 2017 in Germany [36]. Moreover, in recent years, PPIs became available as over-the-counter drugs. Since 2016, however, the prescription numbers have been declining, possibly because evidence has accumulated suggesting that long-term use of PPI may be associated with adverse health effects including dementia [8, 9, 26, 37].

Currently, there is no consensus on the association between the use of PPIs and the risk of dementia [5–7]. Inconsistencies between observational studies, especially those based on claims data, have contributed to the doubtfulness of their utility in clinical decision-making [38, 39]. Specifically, summary effect estimates of several recent meta-analyses suggested no effect of PPI use on dementia risk [5, 6, 40, 41]. On the other hand, plausible pathophysiological pathways of brain deterioration that PPIs might be involved in such as increased amyloid-β plaques, increased tau protein formation, and vitamin B12 deficiency have been described and need to be taken into account when evaluating the available evidence [4].

While we found no evidence for an association between PPI use and brain volumes or estimated brain age, different results of the verbal training and memory tests were observed. We found that PPI users had lower VMLT scores, but the effect sizes were small (Cohen’s *d* = 0.13 for immediate memory, 0.17 for delayed recall). There was no difference in both types of recall between PPI users and non-users when the NAI was used for the examination. The difference in the results between the two verbal memory tests might be caused by the difference in the complexity of the tests, i.e., the different numbers of words and the fact that participants only had to distinguish the distractor words for the NAI test, instead of actively recalling the test words. Besides, the participants of the NAI were younger than the ones of the VLMT since the cohort of the SHIP-2, which includes VLMT, was older (see Table 3).

Given that the two verbal learning and memory tests yielded different results, we additionally checked, whether the size of the left hippocampus, which has been shown to be positively associated with verbal memory in previous studies [42, 43], was different in PPI users and non-users. The disagreement of the test results also supports the necessity for further research. Furthermore, the Mini-Mental State Examination (MMSE) and the Montreal Cognitive Assessment (MoCA) are more often used to account for the general cognitive functions and the risk of dementia [44–46]. Thus, it could be advantageous to employ those exams that consider overall cognitive functions, including visuo-spatial processing and executive functions, attention, recall, orientation, abstraction, and language, to assess general cognition impairment or risk of dementia, rather than focusing only on verbal memory. From our study population, only a limited number of individuals at 60 years or older went through the MMSE.

Our study has several strengths. To the best of our knowledge, this is the first study on the relation between PPI use and brain volumes/brain age assessed by MRI. It is important because previous studies showed conflicting results on the association between PPI intake and dementia/cognitive decline since Gomm et al. [8] reported an increased risk of dementia associated with long-term PPI use. Given that a consensus of the results is needed in order to implement evidence-based recommendations into clinical settings, and decreased brain volume can be used as a proxy of dementia [11, 12, 47, 48], our quantitative approach investigating brain volumes and their correlations with PPI intake added further findings to the body of literature. Additionally, IPW was used to decrease the selection bias caused by non-participation at the brain MRI examination.

We also acknowledge the following limitations of the present study. The study had a cross-sectional design, and we cannot be sure that PPI use preceded changes in brain volumes, estimated brain age, and verbal memory tests. In particular, we cannot rule out reverse causation (i.e., cognitive decline or dementia may predispose to gastric problems and PPI intake). PPI intake was defined as reported regular daily intake over the past 7 days. Unfortunately, detailed information on the duration of intake was unavailable. Lumping short-term and long-term intake into one exposure group might have introduced misclassification and might have biased the effect estimate towards the null (i.e., based the true effect of long-term PPI intake on cognition and brain age/volumes).We cannot also rule out prevalent user bias that could have attenuated true effect sizes. Also, there is a chance that those who irregularly took PPIs or participants with prodromal dementia underreported PPI intake.

Regarding the outcome evaluated, we could not conduct further examinations to diagnose dementia, such as positron emission tomography scans or more specific cognitive tests. Furthermore, white matter hyperintensities, which indicate cerebral small vessel disease [49] and might be associated with PPI intake, could not be precisely quantified by this method. Another limitation is that we were not able to make a direct comparison of the results between VLMT and NAI.

In conclusion, our findings did not support previous evidence on a possible association between PPI intake and brain aging. Further longitudinal investigations of the association between incident PPI use and change in brain volumes and brain aging are needed to confirm this finding.

## Supplementary information

ESM 1 (PDF 349 kb).

## Data Availability

All the datasets created are reported in this manuscript.

## Abbreviations

PPIs: Proton pump inhibitors
SHIP: Study of Health in Pomerania
VLMT: Verbal Learning and Memory Test
NAI: Nuremberg Age Inventory
IPW: Inverse Probability Weighting
