# Supplementary material for: Lack of association between proton pump inhibitor use and brain aging: a cross-sectional study
Source: Eur J Clin Pharmacol. 2021 Jan 13;77(7):1039–48. doi: 10.1007/s00228-020-03068-8 (PMC8184524; doi:10.1007/s00228-020-03068-8)
Supplement: Supplementary file 1 — (PDF 349 kb). [file 228_2020_3068_MOESM1_ESM.pdf]

Supplementary material

Table 1S. Brain lesions and vascular risk factors that might affect brain volumes

**Brain lesions (n= 679)**	**Vascular risk factors (n= 27)**
Acute intracranial ischemia	Vascular occlusion in the head
Intracranial bleeding	Vascular occlusion of the internal carotid artery in the head
Subdural hematoma / hygroma	Vascular occlusion of the cerebral artery in the head
Epidural hematoma	Vascular occlusion of the posterior cerebral artery in the head
Subarachnoid hemorrhage	Anterior cerebral artery occlusion in the head
Parenchymal defect	Vascular occlusion of the basilar artery
Unclear edema in the head	Stenosis> 50% in the head
Mass or Tumor in the head	Stenosis> 50% of the internal carotid artery in the head
Center line shift in the head	Stenosis> 50% of the cerebral artery in the head
Cyst in the head	Stenosis> 50% of the posterior cerebral artery in the head
Vascular abnormalities in the head	Stenosis> 50% of the anterior cerebral artery in the head
Aneurysm in the head	Stenosis> 50% of the basilar artery in the head
Arteriovenous malformation in the head	Stenosis> 50% of the vertebral artery in the head
Developmental venous anomaly in the head	
Malformation in the head	
Cerebellar tonsil depression	
Abnormalities in the pituitary gland	

Table 2S. Linear regression estimates for the associations of PPI intake with brain volumes, brain age and verbal memory assessment by applying inverse probability of treatment weighting (IPTW)

	Coefficient	SE	95% CI	*P*
GMV	0.68	7.54	(-14.10, 15.46)	.93
WMV	-2.41	7.91	(-17.90, 13.10)	.76
TBV	-1.72	14.53	(-30.20, 26.76)	.91
HV	-0.04	0.09	(-0.21, 0.13)	.64
Left HV	-0.02	0.04	(-0.11, 0.06)	.55
Right HV	-0.01	0.05	(-0.11, 0.08)	.75
Brain age	1.27	1.38	(-1.43, 3.96)	.36
VLMT- Immediate recall	-0.84	0.67	(-2.19, 0.50)	.22
VLMT- Delayed recall	-0.75	0.32	(-1.37, -0.13)	.02
NAI- Immediate recall	-0.14	0.12	(-0.38, 0.10)	.25
NAI- Delayed recall	-0.28	0.15	(-0.57, 0.01)	.06

GMV, Brain gray matter volume (ml); WMV, Brain white matter volume (ml); TBV, Total brain volume (ml); HV, Hippocampal volume (ml) ; VLMT, Verbal learning and memory test (n=1,569); NAI, Nuremberg Age Inventory (n=4,142)

Figure S1. Right hippocampal volume differentiated by PPI intake

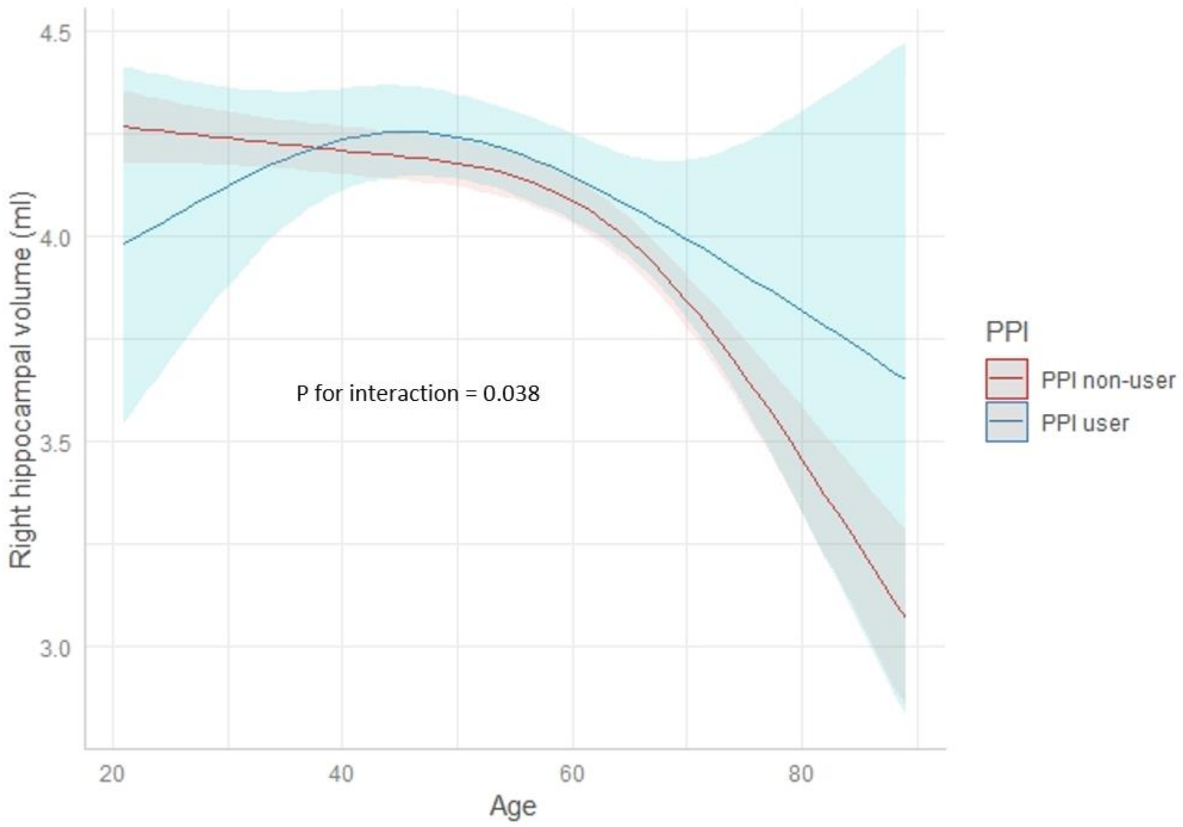

P for interaction between PPI use and age
